# Obstructive Sleep Apnea in Pediatrics and Adolescent Women: A Systematic Review of Sex-Based Differences Between Girls and Boys

**DOI:** 10.3390/children11111376

**Published:** 2024-11-12

**Authors:** Sofía Romero-Peralta, Cristina Rubio, María Castillo-García, Pilar Resano, Miguel Alonso, Esther Solano-Pérez, Laura Silgado, Esther Viejo-Ayuso, Leticia Álvarez-Balado, Olga Mediano

**Affiliations:** 1 Sleep Unit, Pneumology Department, Hospital Universitario de Guadalajara, 19002 Guadalajara, Spain; sofiamp10@hotmail.com (S.R.-P.); cristina.rubio@gmail.com (C.R.); mariacastillogarcia37@gmail.com (M.C.-G.); presanob@gmail.com (P.R.); alonsomiguel23@gmail.com (M.A.); 1399esther@gmail.com (E.S.-P.); guala12@hotmail.com (L.S.); unidadsuenoesther@gmail.com (E.V.-A.); leticia.alvarez.balado@gmail.com (L.Á.-B.); 2 Centro de Investigación Biomédica en Red de Enfermedades Respiratorias (CIBERES), 28029 Madrid, Spain; 3 Medicine Department, Universidad de Alcalá, 28805 Madrid, Spain; 4 Instituto de Investigación Sanitaria de Castilla La Mancha (IDISCAM), 45071 Toledo, Spain

**Keywords:** obstructive sleep apnea, children, sex differences, phenotypes

## Abstract

Background/Objectives: Obstructive sleep apnea (OSA) is marked by repetitive occurrences of upper airway (UA) obstruction during sleep. Morbidities impacting the metabolic, cardiovascular (CV) and neurological systems are correlated with OSA. Only a few studies have described the existence of different characteristics depending on sex and, to date, the girl phenotype in OSA pediatrics is not well known. The objective of this systematic review is to identify the specific phenotype of OSA in pediatric and adolescent females compared to males. Methods: A systematic review was performed. The terms “pediatric sleep apnea” and “sex differences” were used to look for publications using PubMed, the Cochrane Library and Web of Science. Inclusion criteria: (1) peer-reviewed journal articles written in English; (2) investigations conducted on individuals diagnosed with OSA; and (3) investigations providing information about sex differences. Exclusion criteria: (1) studies carried out with individuals aged 18 years and older; (2) studies involving a sample size of fewer than 10 patients; and (3) editorials, letters and case reports. Results: Fifteen studies were included and classified in sections related to sex-based differences. Conclusions: Limited information related to sex-based OSA differences in the pediatric population exists. These differences are conditioned by hormonal status, and are minimal in the premenarcheal period. Moreover, adolescent women present a lower prevalence of obesity and craniofacial alterations, lower OSA severity related to higher UA area and earlier tonsil regression. Hyperactivity is more frequent in boys. Some studies pointed to a higher risk of high diastolic blood pressure in girls than in boys.

## 1. Introduction

One of the primary functions of sleep is to promote well-being, making it an important factor in healthcare promotion. The importance of adequate and quality sleep is supported by scientific research [1,2]. Understanding sex differences in sleep is important for addressing specific consequences in management and related comorbidities.

### 1.1. Sex Differences in Women’s Sleep Along Life

At every stage of a woman’s life, there is an elevated likelihood of experiencing different types of disruptions in sleep, like reduced quality of sleep, and specific sleep disorders such as obstructive sleep apnea (OSA), insomnia or restless legs syndrome (RLS) [3]. Females experience significant changes in sleep patterns starting from neonatal life due to hormonal variations, with notable differences emerging during puberty, menarche, pregnancy and menopause [4]. Most studies on sleep changes in women focus on the alterations that occur during pregnancy and menopause. Pregnancy is characterized by dynamic physiological transformations that influence sleep quality and the frequency of sleep disorders [5] due to anatomical and metabolic changes [6]. During menopause, women experience vasomotor symptoms that worsen the quality of sleep, as well as alterations in their circadian rhythm [7]. These findings confirm that women differ from men in terms of sleep, and thus require a different approach to management.

### 1.2. Sex Differences in Girls’ Sleep

Nowadays, there is limited information about variations in sleep during the initial years of a woman’s life despite the fact that general health sex differences in pediatric and adolescence have become a focus of attention in recent years. Silveyra et al. highlighted the significance of incorporating sex considerations in research studies, also in children [8], and described sex-related differences in respiratory disease from the neonatal stage, including OSA.

During the early years of life, sex-related differences in sleep are minimal. However, these minimal differences can be attributed to the highly dynamic nature of sleep during this period. This variability complicates the study of these differences, leading to contradictory findings across various studies [9,10,11]. Evidence suggests that male infants experience slower maturation of the central nervous system [12].

This lack of important differences persists until puberty and changes secondary to hormonal consequences from menarche. These menstrual hormonal changes influence sleep architecture, with the most significant alteration in the non-rapid eye movement (NREM) phase during the post-ovulatory luteal phase, characterized by elevated electroencephalogram (EEG) activity. This change coincides with higher levels of progesterone and estradiol, as opposed to the follicular phase with lower progesterone levels [13].

Progesterone and testosterone are both influential in regulating respiratory drive, yet they exert opposing effects [14]. Progesterone, commonly elevated in females during certain phases of the menstrual cycle, enhances the sensitivity of the brain’s respiratory centers to carbon dioxide. This effect increases respiratory drive, promoting greater ventilation and potentially lowering the risk of airway obstruction, as seen in some studies on sleep-disordered breathing. In contrast, testosterone, which is present at higher levels in males, may reduce ventilatory response by blunting the sensitivity to carbon dioxide, which can contribute to a predisposition for airway collapse, especially during sleep.

Research suggests that this hormonal influence may help explain the differing prevalence and severity of OSA between males and females, particularly during life stages when hormonal levels fluctuate, such as puberty. Further understanding of these hormonal effects on respiratory function is crucial, as it not only provides insight into the mechanisms behind OSA risk but also raises the potential for hormone-based interventions or considerations in OSA management tailored to sex-based differences.

Additionally, sleep disorders such as insomnia become more prevalent among adolescent females [15]. Xianchen Liu et al. suggest that irregular menstrual cycles and menstrual pain are related with sleep disturbances, and that early menarche (≤11 years old) may temporarily affect sleep patterns in adolescent girls [16].

### 1.3. Obstructive Sleep Apnea (OSA) in Children

Sleep-disordered breathing (SDB) constitutes a comprehensive categorization including a wide spectrum, from snoring to OSA, wherein the latter should be highlighted related to its highly prevalence and important consequences [17]. OSA is defined by recurrent airway obstruction during sleep, leading to episodic hypoxia and disruptions in sleep continuity. Most studies report a prevalence of OSA between 1 and 4%, but it is important to consider that different diagnosis criteria and study methods have been used [18]. OSA can have significant negative effects on children’s health and development, affecting mainly the cardiovascular (CV), metabolic and neurological sphere [19]. Key risk factors leading to the development of OSA in children involve adenotonsillar hypertrophy, obesity and craniofacial abnormalities. The most prevalent nighttime symptoms include snoring, parental observation of nocturnal apneas and enuresis. During the day, the patients can also suffer from hyperactivity and concentration and memory alterations [20]. Complete sleep study in hospital polysomnography (PSG) is the gold standard diagnostic test for OSA [21]. The severity of OSA is determined by the classification of the obstructive apnea–hypopnea index (AHI), obtained from PSG. OSA is considered mild between 1 and 4.9 events per hour, moderate from 5 to 9.9 events per hour and severe if the AHI is 10 or more events per hour [21]. This classification of the severity of OSA is based exclusively on this parameter and not determined by outcomes.

Surgical procedures and weight loss are the most widely extended treatments, reserving CPAP (continuous positive airway pressure) as a second-line option. At least some of the comorbidities associated with OSA would be reversible after surgery. In addressing OSA surgically, it is important to distinguish between different approaches. Adenoidectomy, the removal of the adenoids, is among the most common surgeries performed in children and is often paired with tonsil removal. Tonsillotomy, a procedure that partially removes the tonsils, offers benefits such as reduced postoperative pain, and shorter recovery time compared to the traditional tonsillectomy, where the tonsils are fully removed. However, leaving a portion of the tonsillar tissue may allow for regrowth, which can sometimes necessitate further surgery to manage persistent or recurrent OSA. Numerous systematic reviews [22,23] have found that adenotonsillotomy leads to reduced postoperative morbidity and shorter surgery times compared to adenotonsillectomy, while providing comparable effectiveness in alleviating OSA symptoms. The choice between these procedures depends on the severity and clinical presentation, with the aim of selecting the most appropriate and effective intervention for each patient.

The use of weight loss as a preferred treatment for OSA over CPAP is emphasized, as managing weight effectively may play a crucial role in treating obesity-related OSA in children and adolescents [24,25]. Also, nasal corticosteroid sprays are an effective option for managing OSA in children and adolescents, particularly as a second-line option before considering surgery, CPAP or orthodontic treatments [26]. These sprays reduce inflammation and improve airflow, especially in cases linked to nasal obstruction or allergic rhinitis. This non-invasive approach can be beneficial in reducing symptoms, potentially avoiding the need for more complex interventions. An allergological evaluation is crucial to identify and address allergic triggers that may contribute to airway inflammation, enhancing the effectiveness of nasal corticosteroids.

### 1.4. OSA Children and Adolescents’ Women-Specific Phenotype

Among adults, multiple investigations have been carried out to deepen our insights into the variations in OSA between sexes. These studies specifically explore nuances in the structure and function of the airway [27,28], clinical presentation [29,30,31,32], polysomnographic features [33,34], comorbidities [35,36] and use of health services [37]. Furthermore, some studies suggest the presence of a specific phenotype of OSA in adult women [38]. However, this phenotype has not been completely described in girls yet. Identifying the different clinical phenotypes in pediatrics and adolescents, just as the identification has been extended in adults, could be a strategy to improve the underdiagnosis of OSA in children. The available evidence indicates that OSA is a multifaceted disorder that is recognized by a significant range of patient responses to treatment and health outcomes. While there is an increasing amount of scientific knowledge regarding the phenotyping of OSA in adults, comparable efforts for pediatric OSA remain relatively scarce.

Within this review, we will selectively pinpoint contentious aspects in pediatric OSA, exploring dimensions of general characteristics, clinical manifestations, polysomnographic findings, consequences and treatment. Particular emphasis will be placed on understanding sex differences, with the goal of identifying and analyzing these variations to improve early diagnosis and develop tailored treatment strategies that reduce adverse effects and related comorbidities. The objective of this review is to identify the specific phenotype of OSA in pediatric and adolescent females compared to males.

## 2. Materials and Methods

### 2.1. Search Strategies

This systematic review was designed using the Preferred Reporting Items for Systematic Reviews and Meta-Analyses (PRISMA) (see Supplementary Materials). The search strategy was developed using PubMed, the Cochrane Library and Web of Science, aiming to identify all articles that contribute insights into the link between pediatric OSA and sex. The terms “pediatric sleep apnea” and “sex differences” and age filter (under 18 years) were utilized to identify relevant studies. A comprehensive literature search was conducted from 1982 to 31 January 2024, and the last consultation of sources was in February 2024. The search strategy was consistent across all sources.

### 2.2. Study Selection

We included studies that met the following inclusion criteria: (1) peer-reviewed journal articles written in English; (2) investigations conducted on individuals diagnosed with OSA; and (3) investigations providing information about sex differences. We excluded studies that met the following exclusion criteria: (1) studies carried out in individuals aged 18 years and older; (2) studies involving a sample size of fewer than 10 patients; and (3) editorials, letters and case reports. After the initial search for articles, a selection process was carried out in which each article found was checked against the pre-specified eligibility criteria. The process of screening and data abstraction was carried out in two consecutive stages, first by evaluating the title and abstract, and then the full text of each article. Once the included studies had been selected, the characteristics of the studies and the results of interest were extracted. This process was carried out by two of the authors of the review, independently and blinded to the work of the other reviewer, in order to contrast and reduce the risk of errors during the process.

### 2.3. Data Collection and Risk of Bias

**Figure 1:**
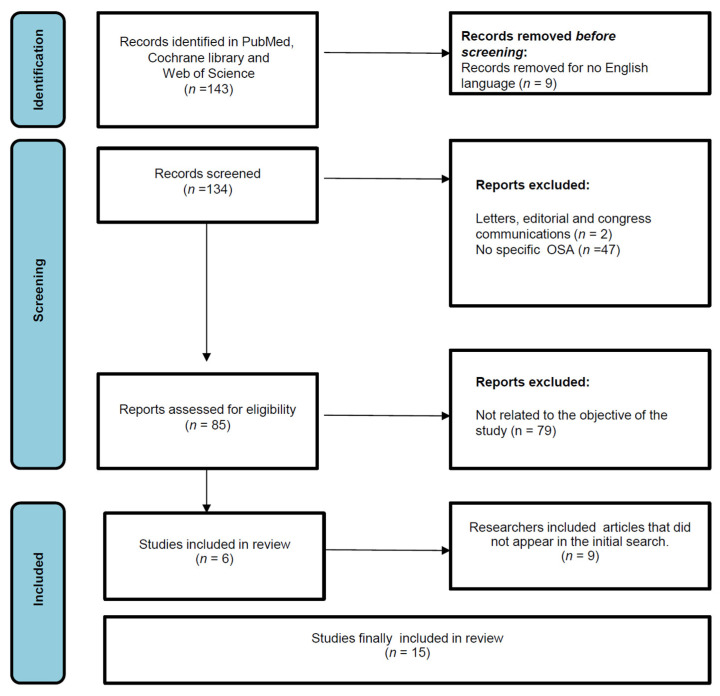
Study selection flow chart.

Final data collection was completed using an electronic database. Two reviewers independently conducted the extraction process of the data collected and included the main sex difference outcomes related to pediatric OSA. For each of the selected studies, the title, first author, publication year, the type of study, objectives, sample size, median age, percentage of boys and girls in the study, the sleep test used for diagnosis OSA and the main findings related to sex differences in OSA were detailed. Upon the initial assessment of the literature, 143 studies were identified. Nine records were removed before screening because they were not in the English language. Following an initial process of screening analyzing the titles and abstracts based on predefined inclusion criteria, 49 articles were excluded, of which 47 studies lacked information on OSA, and 2 were letters, editorial or case reports. Eighty-five studies were assessed for eligibility. A comprehensive evaluation of 85 articles was performed, leading to the exclusion of 79 articles due to their lack of relevance to the study’s objectives. Finally, 6 studies were included in review, and the researchers incorporated 9 additional articles, initially absent from the initial search. These articles were included after carrying out an analysis of the bibliography included in the articles resulting from the initial search, based on their relevance and connection to the review. Ultimately, 15 studies with adequate data were integrated into the narrative synthesis for the present review. The study selection and data collection ‘flow chart’ graphically expresses how the selection of articles was carried out (Figure 1).

Two reviewers independently assessed the risk of bias of each research using the Quality in Prognosis Studies tool recommended by Cochrane.

### 2.4. Synthesis Methods Used

This review employs an inclusive methodology and narrative style to comprehensively examine previous accomplishments of sex differences in children with OSA.

## 3. Results

### 3.1. Sex Differences in General Characteristics

#### 3.1.1. Prevalence and Incidence

Certain studies propose that the prevalence of differences between sexes is not observed in the initial years of life, while disparity emerges after the onset of puberty [39]. In this prevalence of disparities between sexes, age and sexual maturation would be determinant. Kang KT et al. [39], in a total of 1842 children, found that AHI gradually increased with age, but boys had a higher AHI than girls in all age groups (7.8 vs. 4.1 events/h, *p* < 0.001). The average AHI for the girls remained consistent across the different age groups (*p* = 0.492). When analyzing the progression over a 5-year follow-up period, adolescent boys were more likely to develop OSA compared to girls. Additionally, over one-third of the boys who did not have OSA at baseline developed OSA during the course of the study [40].

However, the NANOS study identified that the prevalence of OSA in a prepubertal obese community was similar in girls (42.5%) and boys (37%) [41]. In contrast, research by Selvadurai et al. [42] reported significant differences in a pubertal obese cohort, with OSA being significantly more prevalent in males (*p* = 0.002).

When obesity was present in the population of Kang KT et al. [39], boys also had a higher AHI than girls (14.7 vs. 7.8 events/h, *p* = 0.005).

#### 3.1.2. Physical Examination

There appears to be a lack of research demonstrating sex-based variations in the prevalence of adenotonsillar hypertrophy in adolescents. Inhosita et al. [43] found that adolescent boys exhibited a statistically significant reduction in upper airway (UA) area and increased adenoid size compared to their female counterparts, as observed by lateral radiography. However, in the preadolescent group, no substantial differences in radiographic measurements were reported between boys and girls. Katz et al. [44] collected neck circumference (NC) and body mass index (BMI) data from 245 children aged 6–17 years. An increased risk of OSA was observed in children whose NC was greater than the 95th percentile for their age and sex. When examined by sex, among males ≥ 12 years, an NC exceeding the 95th percentile was significantly linked to an increased risk of OSA (relative risk 3.3, *p* = 0.04). However, this association was not observed in females (*p* = 0.63). These results were not reproducible in BMI or waist circumference > 95th. In addition, Selvadurai et al. [42] described that females presented a higher hip circumference (*p* = 0.04) compared to males. In the same study, a significant interaction between male sex and waist-to height ratio was found.

Di Francesco et al. [45] correlated OSA with craniofacial features and facial patterns according to sex in 77 children (3–12 years old), and found that the cephalometric analysis significantly correlated with the AHI in boys but not in girls.

### 3.2. Sex Differences in Clinical Manifestations

Selvadurai et al. [42] included children aged 8 to 18 years diagnosed with both obesity and OSA. In the male group with OSA, there was a notable increase in reported difficulties with breathing (*p* = 0.04) and a higher prevalence of mouth breathing (*p* = 0.008) compared to females with OSA.

In a study that considered both age and sex, it was observed that the prevalence of snoring was notably higher among boys aged 11 to 13 compared to both girls and boys aged 5 to 10 years [46].

### 3.3. Sex Differences in Polysomnographic Findings

Currently, there are few publications on the analysis of PSG data in boys and girls, and the differences observed in PSG are still being studied. OSA females showed a longer sleep latency onset (45.8 ± 40.6 min vs. 22.4 ± 26.7; *p* = 0.02) [42]. However, in terms of sleep phase distribution [47], the percentage of N1, N2, N3 and rapid eye movement (REM) in girls was similar to those in boys. Previous studies revealed variations in the severity of OSA among adolescent patients based on sex, while others did not identify such sex differences. In some studies, it has been concluded that boys have a higher AHI than girls in the age groups 3–18 years [35]. In addition, it has been observed that during adolescence, girls have a significantly lower AHI and ODI of 3%, together with higher oxygen saturation and sleep efficiency than boys [43]. However, Horne et al. study [47] reported no sex differences in the severity of SDB in children between 3 and 18 years.

The results on specific OSA patterns in a previous study showed that females presented a higher supine AHI (32.9 ± 31.1 vs. 20.4 ± 18.4 events/hour; *p* = 0.02) compared to males [42]. Additionally, according to Chamnanpet et al. [48], females were more likely to have OSA related to REM (*p* = 0.042).

### 3.4. Sex Differences in OSA Consequences and Treatment

#### 3.4.1. Cardiovascular

Horne et al. [47] studied a total of 298 boys and 235 girls with ages between 3 and 18 years old and found no significant sex differences in systolic blood pressure (BP) or the proportion of children with hypertension between any of the SDB severity groups.

However, diastolic BP was elevated in girls suffering from moderate-to-severe OSA (*p* < 0.05), while a smaller number of boys were classified as pre-hypertensive within the moderate-to-severe OSA groups (*p* < 0.05).

#### 3.4.2. Neurocognitive Function, Mood and Behavior Problems

Wu et al. [49], in a retrospective study of 298 boys and 139 girls with OSA, described that boys were more likely to develop attention-deficit/hyperactivity disorder than girls with OSA. Also, Chang et al. [50], in a 15-year retrospective cohort study based on a population sample, reported a significantly elevated risk of later depressive disorders among children diagnosed with OSA, especially among boys and those aged 12 years or older (hazard ratio (HR) = 7.1; *p* = 0.0004). In the findings by Horne et al. [47], girls with moderate–severe OSA exhibited a higher prevalence of internalizing behavioral problems, which include mood disturbances such as anxiety, depression and social withdrawal, in comparison to boys with the same condition (59.2 ± 2.4 vs. 51.4 ± 2.3, *p* < 0.05). Stratifying the data further by age, girls over the age of 9 with moderate-to-severe OSA had significantly worse scores on quality of life and behavioral and executive functioning compared with men and controls of the same age without sleep apnea. This suggests that older females with moderate–severe OSA are particularly vulnerable to these adverse effects.

#### 3.4.3. Sex Differences in Treatment

Evidence specifically addressing sex differences in response to surgery in children with OSA and tonsillar hypertrophy is limited. Most studies included both male and female participants without information comparing both sexes. Spilsbury et al. reported that in children with severe or moderate OSA, boys showed a greater risk of persistent OSA [51]. In a 1-year retrospective study of CPAP adherence, being female was associated with higher adherence rates (60.9% vs. 39.5%; odds ratio (OR) = 2.41, 95%; *p* = 0.01) [52].

The results are summarized in Table 1 and Table 2.

## 4. Discussion

This is a systematic review addressing sex differences and their associated risks, attending the need for research on sex differences in OSA in children. Such knowledge is crucial for early detection and management, aiming to prevent adverse effects and associated comorbidities.

An important contribution of this study is its comprehensive review of the literature on sex differences in pediatric OSA. It is one of the few systematic reviews available on this topic that offers an exhaustive overview of the existing knowledge, highlighting key findings and gaps in the literature.

In the pediatric and adolescent population, the discrepancy in the prevalence of OSA depending on sex has not been fully clarified yet. Lumeng and Chervin [18] conducted an epidemiological analysis of SDB with 15 studies indicating a higher rate of OSA among boys, while 19 studies found no significant differences between sexes, highlighting that the majority of participants in these studies were prepubertal children. Certain studies propose that a disparity in OSA between sexes emerges in children after the onset of puberty, while such a distinction is not observed in the initial years of life [41]. These differences during puberty, which persists in adulthood, have been related to physical differences: the distribution of fluids around the neck [54] and anatomical differences in the UA between sexes [55]. Also, hormonal differences between boys and girls can significantly influence the prevalence and severity of OSA. Testosterone, which is more predominant in males, may reduce airway muscle tone, leading to increased airway collapsibility during sleep. This reduced airway stability can exacerbate OSA symptoms in boys, particularly as they enter adolescence and as testosterone levels rise. In contrast, progesterone, which is higher in females, is known to stimulate respiratory drive by increasing the sensitivity of the brain’s respiratory centers to carbon dioxide. This effect can enhance airway stability and reduce the likelihood of airway obstruction, potentially offering a protective effect against OSA in girls. Furthermore, progesterone has been observed to increase UA muscle tone, with higher muscle ventilation stimulation responses to hypoxia and hypercapnia via the chemoreceptor [56], which might contribute to the lower incidence and severity of OSA seen in girls, especially before puberty.

These hormonal mechanisms highlight the clinical relevance of understanding sex differences in pediatric OSA. Acknowledging these differences can guide tailored approaches to diagnosis and treatment, as boys and girls may respond differently to therapies based on underlying hormonal influences.

These findings might provide insight into the variations in OSA severity among males and females, which is more pronounced in adolescents than in preadolescents. In addition, the differences observed may be related to the regression of adenotonsillar hypertrophy in girls and the influence of estrogen, which may be associated with this regression [43,57].

Regarding other findings of the physical examination in children with OSA, it is more frequent to find obesity in boys than in girls [58]. The growing rates of obesity in children seem to correlate with a higher prevalence of OSA among this population. Potential pathophysiological mechanisms involved in this relationship include adenotonsillar hypertrophy due to greater somatic growth, increased critical airway closing pressure, changes in chest wall mechanics, and disruptions in ventilatory control [59]. Nevertheless, the precise interactions and details of these mechanisms have yet to be fully understood. Additionally, fat distribution patterns in children undergo significant changes after puberty, with boys more likely to develop an ‘android’ fat distribution characterized by fat accumulation around the neck and upper body. This pattern can directly impact airway patency, increasing the risk and severity of OSA. This suggests that treating OSA in girls with hygiene and dietary measures may be less effective than in boys. Furthermore, obesity may be the reason why boys have a higher risk of persistent OSA after surgery than girls as reported by Spilsbury et al. [51]. Also, Merikangas et al. [60] described the stronger relationship between obesity and depression in adolescents, and found that obesity is more significantly related to depression in male adolescents than in female adolescents. Additionally, some studies suggest that boys with Down syndrome may exhibit higher prevalence and severity of OSA due to factors like fat distribution and airway anatomy [61]. Future studies about how hormonal changes and obesity modulate these sex differences are needed.

Increased time spent in sedentary activities, such as computer use, may reduce physical activity levels, contributing to energy imbalance and weight gain. This could provide a possible explanation for the higher incidence of obesity in boys.

Studies have reported the existence of two phenotypes of OSA in children [62]: (I) a phenotype related to adenotonsillar hypertrophy, being more common in younger children with similar prevalence between sexes, and (II) a phenotype related to the presence of obesity, which is more common in adolescents, with a higher prevalence in boys than in girls, sharing similarities with adults with OSA. However, the phenotype of girls with OSA is still not well defined.

Children with OSA can exhibit a wide range of symptoms and, to date, there are only a few publications that differentiate between sexes. Data on the symptoms for children are based on questionnaires collected by their parents, so there may be some bias [63], with snoring being the most common symptom reported. Physiopathological factors may indeed explain why snoring and breathing difficulties appear more frequently in boys than in girls with pediatric OSA. Anatomical differences contribute to increased fat accumulation around the neck and upper airway, potentially leading to a narrower airway and greater resistance during sleep. Hormonal influences also play a role; testosterone, more prominent in boys, may reduce airway muscle tone, exacerbating airway collapsibility. Additionally, boys with OSA often exhibit more severe forms of obesity and higher BMI, which are linked to worsened airway patency and a greater likelihood of snoring and respiratory challenges. Less frequent symptoms include paradoxical breathing, gasping, restless sleep, nightmares, frequent awakening, behavioral changes and hyperactivity or aggressiveness. It is only when parents identify these symptoms that clinical advice is sought, leading to the necessary diagnostic tests and procedures, which may introduce a bias based on parental observation. To date, it is not known whether there is a social stigma, as occurring in adults with OSA, to report snoring in young girls versus boys. This raises questions about whether snoring is viewed differently in boys versus girls, potentially influencing parental reporting to avoid stigmatization in girls or perceived family embarrassment. Factors such as snoring volume, frequency and the parents’ monitoring habits may vary by gender, which could impact how often snoring is reported. These biases should be considered when interpreting epidemiologic studies on OSA prevalence, especially since most rely on parent-completed questionnaires or selection criteria potentially influenced by such biases. To date, no studies have looked at whether the symptoms that lead to a doctor’s visit differ between boys and girls. Additionally, the inclusion criteria may influence referral patterns, as differences in symptom presentation, parental perception and possible societal biases could affect when boys or girls are referred for medical evaluation for snoring.

Consequently, this may impact the interpretation of symptom prevalence, severity and progression of OSA between boys and girls, potentially leading to an under- or overestimation of the condition’s characteristics in each group.

Related to respiratory parameters, in the preadolescent group, no distinctions were noted between boys and girls. Interestingly, adolescent girls exhibited notably lower AHI and 3% ODI, along with higher minimum oxygen saturation (SpO_2_) and better sleep efficiency compared to boys. These findings suggest that variations in OSA severity related to sex may be more pronounced or exclusively evident during adolescence [43], even in obese populations, where it has been described that boys may have a higher AHI than girls [39].

According to CV morbidities associated with OSA, they encompass systemic hypertension, heightened sympathetic activation, and ventricular hypertrophy. Moreover, in severe cases of OSA, infants and children may occasionally experience pulmonary hypertension and right heart failure [64]. However, these CV diseases are very similar between boys and girls, except for one study reporting higher diastolic BP in females [47].

More differences have been reported in the neurological sphere. In children, behavioral alterations (areas of attention, learning, social skills), neurocognitive deficits or problems in verbal and non-verbal learning and alterations in school performance [65,66] are more frequently described. In some publications, cognitive deficits are related to the severity of OSA in a dose-dependent manner, although it is also described in mild OSA [67]. Boys seem to more frequently present hyperactive symptoms than girls [49,53] and a tendency to depressive symptoms [50,68]. Females with moderate–severe OSA showed a higher incidence of internalizing behavioral problems compared to males [47].

In relation to treatment response, there are not enough data in the literature review that differentiate it according to the child’s sex. Middle-childhood and late-adolescent males have been associated with a higher persistent OSA after treatment [51]. In this case, an early intervention for preadolescent boys could be considered, but watchful waiting could be enough for girls.

The higher likelihood of obesity in boys with OSA suggests that a greater proportion of them might be treated first with weight loss and later with CPAP compared to girls with OSA. However, there are no data to support this. Only one study has reported a higher adherence to CPAP treatment in girls than in boys [52].

Other alternative treatments have been proposed to treat OSA in children such as nasal corticosteroid sprays, myofunctional therapy [69] or rapid maxillary expansion [70], but the differences in response to these treatments according to the sex of the children have not been studied either.

More studies are needed to determine the differences between boys and girls with OSA from a comprehensive point of view to define the specific phenotype in girls with OSA. A better understanding of the sex differences can provide exciting prospects to optimize the management of pediatric OSA and its outcomes, facilitating more tailored approaches for diagnosing and treating children with OSA.

Finally, the strengths and limitations of this review should be critically evaluated. The strengths of this study include a thorough review of the existing literature of sex differences in pediatric OSA, providing a comprehensive overview of current knowledge in the field. Secondly, this study highlights an important gap in the literature considering sex-specific factors in understanding OSA, suggesting an extensive investigation of this topic, which may be considered for clinical practice and personalized management. Limitations of this study involve, principally, the scarcity of the existing literature, which may limit the interpretation and conclusions that can be drawn from the systematic review. We acknowledge that language restrictions may have limited the inclusion of certain studies relevant to the topic, potentially narrowing the scope of findings and excluding valuable insights published in non-English languages. In addition, variability in the methodology between the studies (mainly in the type of sleep study and OSA stratification), outcome measurements and cohort characteristics may introduce challenges in generalizing findings. Finally, the scarcity of longitudinal studies may restrict the ability to evaluate long-term consequences of sex differences in pediatric OSA.

## 5. Future Research

Further studies are needed to demonstrate whether there are indeed differences between OSA in girls and boys, and whether these differences have clinical relevance for its management in the pediatric population. Studies are needed to examine the role of the following:

1. Hormonal Influence on OSA: Future studies should investigate hormonal effects, especially during puberty, on OSA severity in boys and girls. Hormones like progesterone and estrogen might provide girls with some protective effect on airway tone.
2. Sex-Specific OSA Phenotypes: Research should focus on defining distinct OSA phenotypes based on sex, factoring in obesity, fat distribution and upper airway anatomy. Girls may exhibit a less severe phenotype with higher rates of adenotonsillar regression compared to boys.
3. Lifestyle, Obesity and Sedentary Behavior: Studies should examine how obesity and physical inactivity affect OSA risk and severity in boys and girls differently. Boys may experience more persistent OSA due to higher obesity rates and android fat distribution.
4. Cognitive and Behavioral Outcomes: More research is needed on the neurocognitive and behavioral impacts of OSA in each sex, particularly regarding attention, learning and emotional regulation. Boys may be more prone to hyperactivity, while girls may have internalizing symptoms.
5. Treatment Efficacy and Adherence: Evaluating differences in CPAP, surgery and alternative treatments based on sex could inform tailored protocols. Girls may show higher adherence to CPAP.
6. Longitudinal Studies on OSA Progression: Long-term studies are essential to understand how OSA severity, comorbidities and treatment outcomes evolve based on sex from childhood through adolescence, potentially improving early intervention approaches.
7. High-Risk Groups: High-risk groups, such as children with Down syndrome, also warrant further study to determine whether sex differences in OSA presentation and severity exist within this population.

## 6. Conclusions

There exists limited information related to sex-based OSA in the pediatric population. These differences are mainly conditioned by hormonal status, and are minimal in the premenarcheal period. Additionally, adolescent women present a lower prevalence of obesity, lower OSA severity related to higher UA and earlier tonsil regression. Hyperactivity and depressive symptoms are more frequent in boys. Without important differences in associated CV risk, some studies pointed to a higher risk of high diastolic blood pressure in girls than boys.

## Figures and Tables

**Table 1:** Main sex differences in polysomnographic findings in children with OSA.

Reference	Type of Study	OSA Diagnosis	Main Findings
Ersu R et al. 2004 [46]	Observational, longitudinal study 2147 children (51% M) Aged 5–13 yr (mean 8.5 ± 1.3 yr)	Questionnaire about sleep habits, nocturnal and daytime symptoms and medical history High risk of OSA: apnea and difficulty breathing in addition to snoring.	Clinical manifestations: Significantly higher prevalence of snoring in M than F (8.5% vs. 5.6%, respectively; *p* = 0.008). Specifically, snoring prevalence was significantly higher in M > 11 yrs.
Goodwin JL et al. 2010 TuCASA study [40]	Prospective cohort study *n* = 480 (50.4% M) 6–11 yr (mean 8.5 yr)	Study test: PSG SDB cutoff: RDI ≥ 1/h associated with a ≥ 3% OD	Prevalence: In a cohort with a 5-year follow-up, boys were more likely to develop SDB (OR = 3.93; *p* = 0.008). Children with prevalent SDB were more likely to be boys (OR = 2.48; *p* = 0.006).
Di Francesco et al. 2012 [45]	Prospective study *n* = 77 (62% M) Age 3–12 yr	Study test: PSG OSA cutoff: AHI ≥ 1/h	Prevalence: No statistical difference in the prevalence of sleep apnea was observed between boys and girls (*p* = 0.463). Physical examination: Cephalometric analysis correlated significantly with the AHI in M but not in F. Sleep and respiratory parameters: M had higher AHI than F (*p* = 0.0406).
Alonso-Alvarez, M.L et al. 2014 [41] NANOS study	A cross-sectional, prospective, multicenter study *n* = 248 obese children (54.4% M) 3–14 yr (mean 10.8 ± 2.6 yr)	Sleep test: PSG OSA cutoff: SRDI ≥ 3/h TST	Prevalence: The prevalence of OSA was similar in girls (42.5%) and in boys (37%; *p* = 0.434). The M:F ratio for either habitual snoring or OSA was slightly higher in prepubertal ages, but significantly increased with ages traditionally corresponding to puberty or post-puberty.
Katz et al.2015 [44]	Population-based dataset *n* = 245 (56% M) 6–17 yr (median 11.7 (6.0–17.9))	Study test: PSG OSA cutoff: AHI > 5/h or OAHI ≥ 1/h	Physical examination: The association of NC > 95th percentile and risk of OSA was significant in males ≥ 12 yr (relative risk = 3.3; *p* = 0.04) but not in females (*p* = 0.63).
Spilsbury JC et al. 2015 [51]	Observational, longitudinal study *n* = 490 (51% M) 8–11 (mean 9.5 ± 0.5 yr) and 16–19 yr (mean 17.7 ± 0.4 yr)	Sleep test: In-home sleep apnea monitoring Type III (middle childhood) and PSG (late adolescent) OSA cutoff: OAHI ≥ 5/h or OAI ≥ 1/h	Response to treatment: In children with moderate and severe OSA, males showed a greater risk of persistent OSA.
Hawkins SM et al. 2016 [52]	Retrospective study 140 children (54% M)	-	Response to treatment: F was associated with better adherence to CPAP than M (60.9% vs. 39.5%; OR = 2.41; *p* = 0.01).
Wu J et al. 2017 [49]	Observational, retrospective study *n* = 437 (68% M) 4–11 yr (mean 5.71 ± 1.45 yr)	Sleep test: PSG OSA cutoff: OAHI > 1/h or AHI > 5/h and lowest SpO_2_ is <92%	Neurocognitive function, mood and behavior problems: M was more likely to develop attention deficit hyperactivity disorder than F (M/F ratio was 2.56:1 in 4–5 yr group and 1.97:1 in 6–11 yr group).
Chang CH et at. 2017 [50]	Population-based study Retrospective cohort study 6237 children (567 OSA, 67% M) < 18 yr (mean 9.70 ± 4.21 yr)	Sleep test: PSG	Neurocognitive function, mood and behavior problems: Higher risk of depressive disorders in children with OSA, particularly M older than 12 yr (HR = 7.1; *p* = 0.0004).
Inoshita A et al. 2018 [43]	Observational, longitudinal study *n* = 63 (68% M) 3–15 yr Preadolescent group 3–8 yr; adolescent group 9–15 yr	Study test: PSG OSA cutoff: AHI ≥ 1/h	Physical examination: The preadolescent group did not present significant differences between M and F in radiographic parameters. In the adolescent group, F had significantly greater UA area and lower adenoid/nasopharyngeal ratio than M, as observed through lateral radiography. Sleep and respiratory parameters: In the preadolescent group, there were no significant differences in the PSG parameters between the boys and girls. F adolescents had lower AHI, 3% ODI and higher minimum SpO_2_ and better sleep efficiency than M.
Horne RS et al. 2020 [47]	Observational, retrospective study *n* = 533 (56% M) 3–18 yr (mean 7.2 ± 0.1)	Sleep test: PSG OSA cutoff: OAHI > 1/h	Sleep and respiratory parameters: There were no differences in SDB severity between sexes. OAHI was slightly higher in F compared to M in the mild–severe OSA group (15.8 ± 0.7/h vs. 13.6 ± 0.6/h, *p* < 0.05). The percentage of N1, N2, N3 and REM sleep phases in F was similar to M. CV consequences: Diastolic BP was elevated (mean of 4 mmHg) in F with moderate–severe OSA compared to M (*p* < 0.05). Fewer M were pre-hypertensive (*p* < 0.05) in said group. Neurocognitive function, mood and behavior problems: F with moderate–severe OSA exhibited more internalizing behavioral problems compared to males (59.2 ± 2.4 vs. 51.4 ± 2.3; *p* < 0.05).
Matlen et al. 2021 [53]	Cross-sectional analysis *n* = 2,327,104 children (9547 OSA, 57% M) 2–18 yr (8.3 ± 4.7 yr)	Sleep apnea was identified using International Classification of Diseases 10th Revision Clinical Modification codes (USA)	Neurocognitive function, mood and behavior problems: Untreated sleep apnea was associated with an increased risk of lower extremity fractures, while treatment for OSA was associated with improved OR of lower extremity fracture only in M.
Selvadurai et al. 2022 [42]	Cross-sectional study *n* = 148 obese children (61 OSA: 69% M) 8–18 yr	Study test: PSG OSA cutoff: OAHI ≥ 5/h	Prevalence: OSA was more prevalent among M with a 2:1 M/F ratio. Physical examination: F presented a higher hip circumference compared to M (*p* = 0.04). An increase in waist-to-height ratio was associated with higher OAHI in M but not F. Clinical manifestations: In the OSA group, M reported more trouble breathing (*p* = 0.04) and mouth breathing (*p* = 0.008) than F. Sleep and respiratory parameters: OSA F showed longer sleep onset latency (45.8 ± 40.6 min vs. 22.4 ± 26.7; *p* = 0.02) and F had higher supine obstructive AHI (32.9 ± 31.1 vs. 20.4 ± 18.4/h; *p* = 0.02) compared to M. A significant interaction was found between male sex and waist-to-height ratio (*p* = 0.05).
Kang KT et al. 2022 [39]	Observational, retrospective study *n* = 1842 (67% M) 3–18 yr (mean 8.0 ± 3.7 yr)	Sleep test: PSG OSA cutoff: AHI > 1/h	Sleep and respiratory parameters: In all age groups, M had a higher AHI than F (7.8/h vs. 4.1/h, *p* < 0.001). AHI in M increased with age (3–6 to 15–18 yr groups: 7.0–13.6 events/h, respectively; *p* trend < 0.001), whereas the AHI in F was not significantly different between ages (*p* = 0.492). M with obesity had a higher AHI than obese F.
Chamnanpet et al. 2022 [48]	Retrospective cross-sectional study *n* = 366 children (67% M) <18 yr	Sleep test: PSG OSA cutoff: AHI ≥ 1/h	Sleep and respiratory parameters: Children with REM-related OSA were more likely to be F (*p* = 0.042), and had lower prevalence of adenotonsillar hypertrophy (*p* = 0.043) compared with M with other OSA subtypes. F sex was a risk factor for REM-related OSA (OR = 1.874; *p* = 0.009).

Abbreviations: AHI: apnea hypopnea index; BP: blood pressure; CPAP: continuous positive airway pressure; F: female; HR: hazard ratio; M: male; NC: neck circumference; OAHI: obstructive apnea hypopnea index; OD: oxygen desaturation; ODI: oxygen desaturation index; OR: odds ratio; OSA: obstructive sleep apnea; PSG: polysomnography; RDI: respiratory disturbance index; REM: rapid eye movement; SDB: sleep disordered breathing; SpO_2_: oxygen saturation; SRDI: sleep respiratory disturbance index; TST: total sleep time; UA: upper airway.

**Table 2:** Table of sex-based differences in pediatrics with OSA: a comparative analysis.

Parameter	Boys	Girls
OAHI	In the preadolescent group, no distinctions were noted between boys and girls. Adolescent boys often have more severe OSA than girls. In obese populations, boys have a higher OAHI than girls.	In the preadolescent group, no distinctions were noted between boys and girls. Adolescent girls exhibited notably lower OAHI and 3% ODI, along with higher minimum oxygen saturation (SpO_2_). Females presented a higher supine OAHI.
Common symptoms	Snoring. Difficulties with breathing and a higher prevalence of mouth breathing.	Snoring frequency is generally lower in girls compared to boys.
Progression	May worsen with age and weight gain, particularly during puberty.	May be influenced by hormonal changes. Adolescent women present a lower prevalence of obesity and craniofacial alterations, lower OSA severity related to higher UA area and earlier tonsil regression.
Comorbidities	Boys seem to present more frequently hyperactive symptoms than girls.	Girls older than 9 years with moderate–severe OSA exhibited a higher prevalence of internalizing behavioral problems, which include mood disturbances such as anxiety, depression and social withdrawal. Some studies pointed to a higher risk of high diastolic blood pressure in girls than in boys.
Sleep architecture changes	The percentage of N1, N2, N3 and rapid eye movement (REM) in girls was similar to boys.	Girls with OSA showed a longer sleep latency onset.
Response to treatment	Middle-childhood and late-adolescent boys have been associated with a higher persistent OSA after treatment.	Better adherence to CPAP.

Abbreviations: CPAP: continuous positive airway pressure; OAHI: obstructive apnea hypopnea index; ODI: oxygen desaturation index; SpO_2_: oxygen saturation; OSA: obstructive sleep apnea; UA: upper airway.

## Supplementary Materials

The following supporting information can be downloaded at: https://www.mdpi.com/article/10.3390/children11111376/s1, PRISMA 2020 checklist. Reference [71] is cited in Supplementary Materials.

## Abbreviations

Apnea/hypopnea index (AHI); blood pressure (BP); body mass index (BMI); cardiovascular (CV); continuous positive airway pressure (CPAP); electroencephalogram (EEG); hazard ratio (HR); neck circumference (NC); non-rapid eye movement (NREM); obstructive sleep apnea (OSA); odds ratio (OR); oxygen saturation (SpO_2_); oxygen desaturation index (ODI); polysomnography (PSG); preferred Reporting Items for Systematic Reviews and Meta-Analysis (PRISMA); rapid eye movement (REM); restless legs syndrome (RLS); sleep-disordered breathing (SDB); lower oxygen saturation time < 90% (T90); upper airway (UA).
